# Arthroscopic remplissage is safe and effective: clinical and magnetic resonance results at a minimum 3 years of follow-up

**DOI:** 10.1186/s10195-021-00624-5

**Published:** 2022-01-08

**Authors:** Pietro S. Randelli, Riccardo Compagnoni, Simone Radaelli, Mauro B. Gallazzi, Alberto Tassi, Alessandra Menon

**Affiliations:** 1 U.O.C. 1a Clinica Ortopedica, Azienda Socio Sanitaria Territoriale Centro Specialistico Ortopedico Traumatologico Gaetano Pini-CTO, Piazza Cardinal Ferrari 1, 20122 Milan, Italy; 2 Laboratory of Applied Biomechanics, Department of Biomedical Sciences for Health, Università degli studi di Milano, Via Mangiagalli 31, 20133 Milan, Italy; 3 Research Center for Adult and Pediatric Rheumatic Diseases (RECAP-RD), Department of Biomedical Sciences for Health, Università degli studi di Milano, Via Mangiagalli 31, 20133 Milan, Italy; 4 Department of Biomedical, Surgical and Dental Sciences, Università degli studi di Milano, Via della Commenda 10, 20122 Milan, Italy; 5 U.O.C. Week Surgery Di Ortopedia E Traumatologia, Azienda Socio Sanitaria Territoriale Centro Specialistico Ortopedico Traumatologico Gaetano Pini-CTO, Piazza Cardinal Ferrari 1, 20122 Milan, Italy; 6 U.O.C. Radiodiagnostica, Azienda Socio Sanitaria Territoriale Centro Specialistico Ortopedico Traumatologico Gaetano Pini-CTO, Piazza Cardinal Ferrari 1, 20122 Milan, Italy

**Keywords:** Shoulder, Instability, Arthroscopy, Remplissage, Hill–Sachs

## Abstract

**Background:**

Large Hill–Sachs lesions are considered a risk factor for recurrence of instability after arthroscopic Bankart repair alone. The aim of this study was to demonstrate that remplissage is a safe procedure that effectively reduces the risk of recurrent dislocations without causing fatty degeneration of the infraspinatus at medium-term follow-up.

**Methods:**

Patients who underwent arthroscopic Bankart repair and remplissage with a minimum 3 years of follow-up were included. Constant–Murley (CMS), American Shoulder and Elbow Surgeons (ASES), and Walch–Duplay scores were evaluated. Magnetic resonance imaging (MRI) was performed to detect the appearance of fatty infiltration inside the infraspinatus muscle, the percentage of the Hill–Sachs lesion filled by the tendon and its integration, and the onset of rotator cuff tears.

**Results:**

Thirteen patients (14 shoulders) with a mean follow-up of 55.93 (± 18.16) months were enrolled. The Walch–Duplay score was 95.00 [87.25–100.00], with a return to sport rate of 100%. Both the CMS and the ASES indicated excellent results. The affected shoulders showed a statistically significant reduction in active external rotation both with the arm at the side (ER1) and with the arm at 90° of abduction (ER2) (*p* = 0.0005 and *p* = 0.0010, respectively). A reduction in infraspinatus isometric strength was found for both ER1 and ER2, but this reduction was only statistically relevant in ER2 (*p* = 0.0342).

There was a traumatic recurrence of instability in two cases (14.28%). MRI evaluation demonstrated an absence of adipose infiltration in 50% of cases and only a minimal amount in the remaining 50%. In 12 cases (85.72%), the capsulotenodesis completely filled the lesion and good tendon–bone integration was observed.

**Conclusion:**

Arthroscopic remplissage provided successful clinical outcomes without fatty infiltration of the infraspinatus and with good healing of the tissues. The low risk of recurrence was associated with an objective limitation on active external rotation, but this did not influence the patients' daily or sports activities.

*Level of evidence*: Cohort study, level of evidence 3.

## Introduction

Hill–Sachs lesions are posterior-superolateral bone defects of the humeral head that can be observed in 45–70% of first episodes of shoulder dislocation and about 100% of recurrent dislocations [1–3]. These compression fractures result from the impact between the posterosuperior portion of the humeral head and the anteroinferior edge of the scapular glenoid [4].

Burkhart and De Beer [5] defined these lesions as "engaging” when they engage at the level of the anterior glenoid edge during abduction and external rotation movements of the arm, leading to a new shoulder dislocation.

Di Giacomo et al. [6] have recently introduced the concept of the "glenoid track,” which is the contact area between the humeral head and the scapular glenoid during shoulder movement in maximum abduction and external rotation. This concept associates the extent of the glenoid bone defect (bony Bankart) with the location, extent, and depth of the humeral bone defect (Hill–Sachs).

**Fig. 1:**
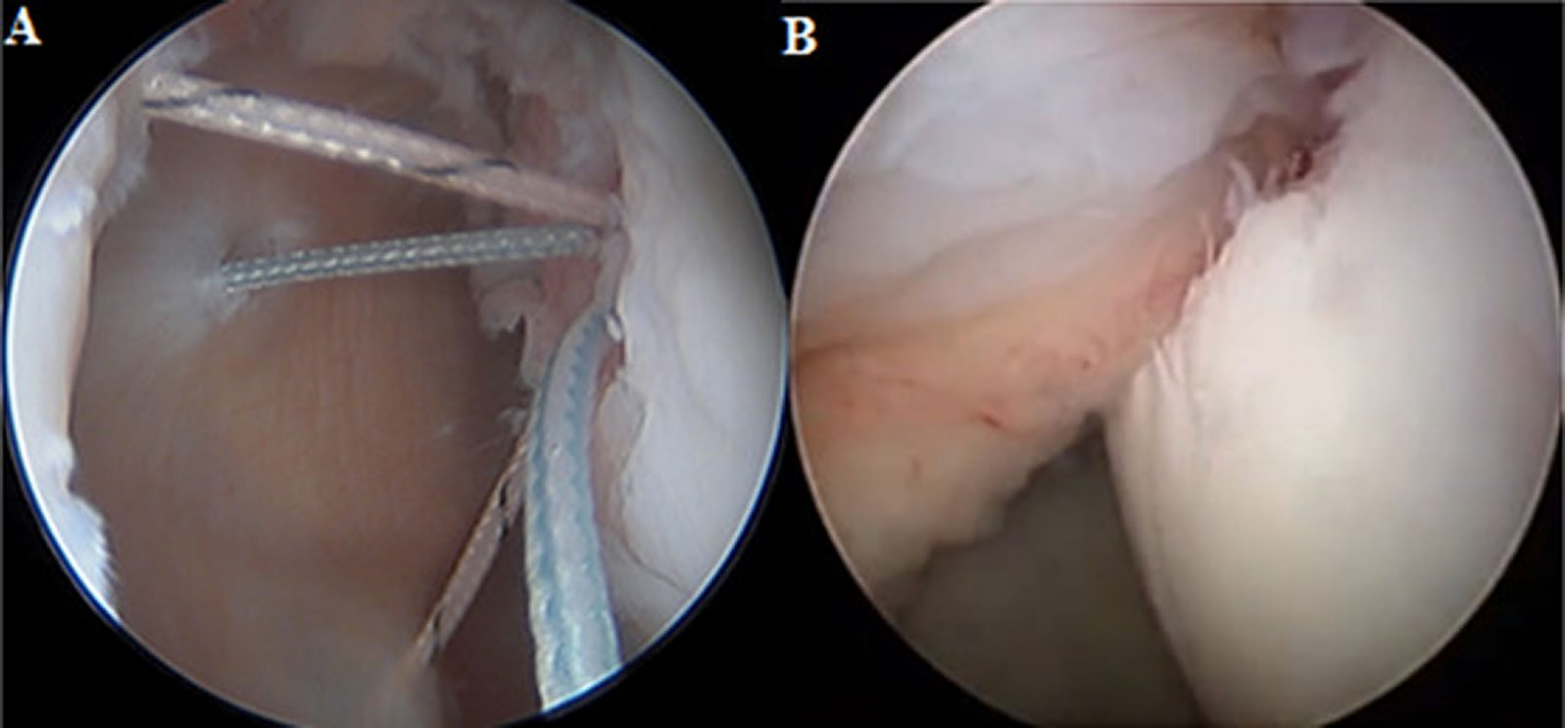
Intra-articular arthroscopic view of a Hill–Sachs lesion. **A** Arthroscopic view of sutures passing through the infraspinatus tendon; **B** the lesion is filled with an infraspinatus tendon capsulotenodesis

An intact glenoid track should guarantee joint stability, but a medial Hill–Sachs can point to a high risk of recurrence if treated only with anterior Bankart repair [6–8]. In 2004, Wolf and Pollack proposed a new procedure for these cases called remplissage, which consists of an arthroscopic capsulotenodesis of the posterior capsule and infraspinatus tendon to "fill" the humeral bone defect. This technique converts the Hill–Sachs from an intra-articular lesion to an extra-articular one, thus preventing its engagement with the anterior portion of the scapular glenoid (Fig. 1) [9]. The remplissage procedure has shown several advantages over the last years, as evidenced by different studies, with minimal complications and restoration of stability in the vast majority of patients [10, 11].

This procedure could potentially reduce the post-surgical range of motion in abduction and external rotation, resulting in pain on the posterosuperior side of the shoulder and leading to degeneration of the infraspinatus tendon and muscle [12, 13].

The present study aimed to analyze the recurrence rate of glenohumeral dislocation and demonstrate if this additional surgical action can lead to fatty degeneration of the infraspinatus at medium-term follow-up.

Based on the fact that Walch and colleagues [14] have shown that moderate fatty infiltration of the involved muscle begins to appear at least 3 years after rotator cuff tears, this study hypothesized that arthroscopic remplissage performed in association with a Bankart repair does not lead to fatty degeneration of the infraspinatus muscle at a minimum follow-up of 3 years.

## Materials and methods

The primary objective of this interventional, monocentric study was to detect the appearance of fatty infiltration in the infraspinatus muscle through the use of magnetic resonance imaging (MRI).

The secondary objectives were to:

- Assess the range of motion (ROM) of the shoulder in external rotation measured with the arm at the side (ER1) and with the arm at 90° of abduction (ER2)
- Measure the infraspinatus isometric strength
- Establish the healing of the capsulotendodesis through the use of MRI
- Quantify the percentage of the Hill–Sachs lesion filled by the tendon, considering the number of anchors used
- Evaluate the presence of tendinopathies, rotator cuff tears, and bone edema of the humeral head.

The Regional Ethical Committee approved this study protocol.

All operations were performed by the same surgeon (PSR) and under general anesthesia with an associated interscalene block. Patients were placed in the lateral decubitus position with the arm maintained at 40° of abduction and 15° of anterior flexion in neutral rotation. A complete arthroscopic examination was performed using a standard posterior portal. A Bankart lesion associated with an “off-track” Hill–Sachs lesion was found in all the patients included in the study. The Hill–Sachs defect was measured with a probe. The Bankart lesion was treated with two absorbable anchors (Lupine 2.9 mm, DePuy Mitek, Raynham, MA, USA), and the Hill–Sachs lesion was treated with one double-loaded metallic suture anchor (Corkscrew 5.0 mm, Arthrex, Naples, FL, USA).

All the patients followed the same postoperative protocol. After the operation, a sling (UltraSling 1, DonJoy Corp., Vista, CA, USA) was used for 25 days. Assisted physiotherapy started 3 weeks after the intervention, and external rotation was limited to 0° until 2 months after the intervention. Gradual muscle strengthening and a gradual return to sporting activities were allowed from 3 months after the operation.

Patients were prospectively evaluated from February 2019 to November 2019 and at least 3 years after surgery, and were assessed for eligibility by a single investigator according to the inclusion and exclusion criteria listed in Table 1.

**Table 1:** Eligibility criteria

Inclusion criteria	Patient is affected by anterior shoulder instability Patient presents an “off-track” Hill–Sachs, confirmed through both preoperative MRI and intraoperative assessment Patient is undergoing arthroscopic Bankart repair in association with Hill–Sachs remplissage Patient has a minimum follow-up of 3 years Patient has a maximum age of 45 years
Exclusion criteria	Patient presents an anterior glenoid bone loss of > 25% Patient is undergoing further surgical procedures on the index shoulder before the postoperative evaluation

At this time, a researcher performed a telephone interview to investigate if the patient had experienced a dislocation recurrence and to collect helpful information for compiling the Walch–Duplay Score [15] and the American Shoulder and Elbow Surgeons Score (ASES) [16]. All patients were invited for a clinical and radiological evaluation. The investigators collected the following data: age, gender, dominant limb, mode of onset of symptoms, type of work activity (light or heavy physical work), preoperative sports activity, and postoperative return to sport.

The authors determined the Constant–Murley Score (CMS) [17] during the clinical evaluation. The active range of motion in abduction (ABD) and external rotation with the arm at the side (ER1) and with the arm at 90° of abduction (ER2) were measured with the use of a goniometer.

The authors measured the isometric strength of the infraspinatus using a stably fixed dynamometer (Kern HCB, Kern & Sohn GmbH, Germany), with the patient in an orthostatic position and the examiner positioned laterally with one hand retracting the scapula. The strength of the affected shoulder and that of the contralateral shoulder were measured in ABD, ER1, and ER2 [18]. One patient had both shoulders operated on; in that case, it was not possible to have a healthy contralateral control, so the side-to-side comparison was carried out on only 12 patients.

Each patient underwent an MRI scan to reconstruct axial, coronal, and sagittal cuts evaluated by the same radiologist, and this MRI was compared to the preoperative one.

The fatty infiltration of the infraspinatus was divided into three degrees according to the staging proposed by Fuchs [19], using the difference in contrast between the adipose tissue signal and the muscular signal.

Based on the method proposed by Park et al. [20], the volume of the Hill–Sachs lesion was measured using a formula for calculating the volume of an ellipsoid and the images acquired in axial and sagittal MRI cuts. Subsequently, the Filling Index Score of Remplissage (FISOR) [21] was evaluated.

A qualitative assessment of the condition of the rotator cuff tendons was carried out, identifying any signs of degeneration or partial or complete tear and then classifying them according to the scale proposed by Snyder [22]. Signs of healing at the tendon–bone interface, the presence of fibrous tissue, and the absence of bone edema were also detected.

### Statistical analysis

The statistical analysis was carried out using GraphPad Prism (v. 6.0; GraphPad Software Inc.) and SAS (v. 9.4; SAS Institute, Inc).

Continuous variables were expressed as the mean ± standard deviation (SD) or the median and first and third quartiles [Q1–Q3], as appropriate. The Shapiro–Wilk normality test was used to evaluate the normality of the sample distribution. For continuous variables, differences between groups of patients were assessed with the unpaired Student’s* t* test or the Mann–Whitney test, according to the characteristics of the data distribution. Categorical variables were expressed in terms of numbers of cases and frequencies. Throughout the analysis, the significance level was set at *p* < 0.05.

## Results

**Fig. 2:**
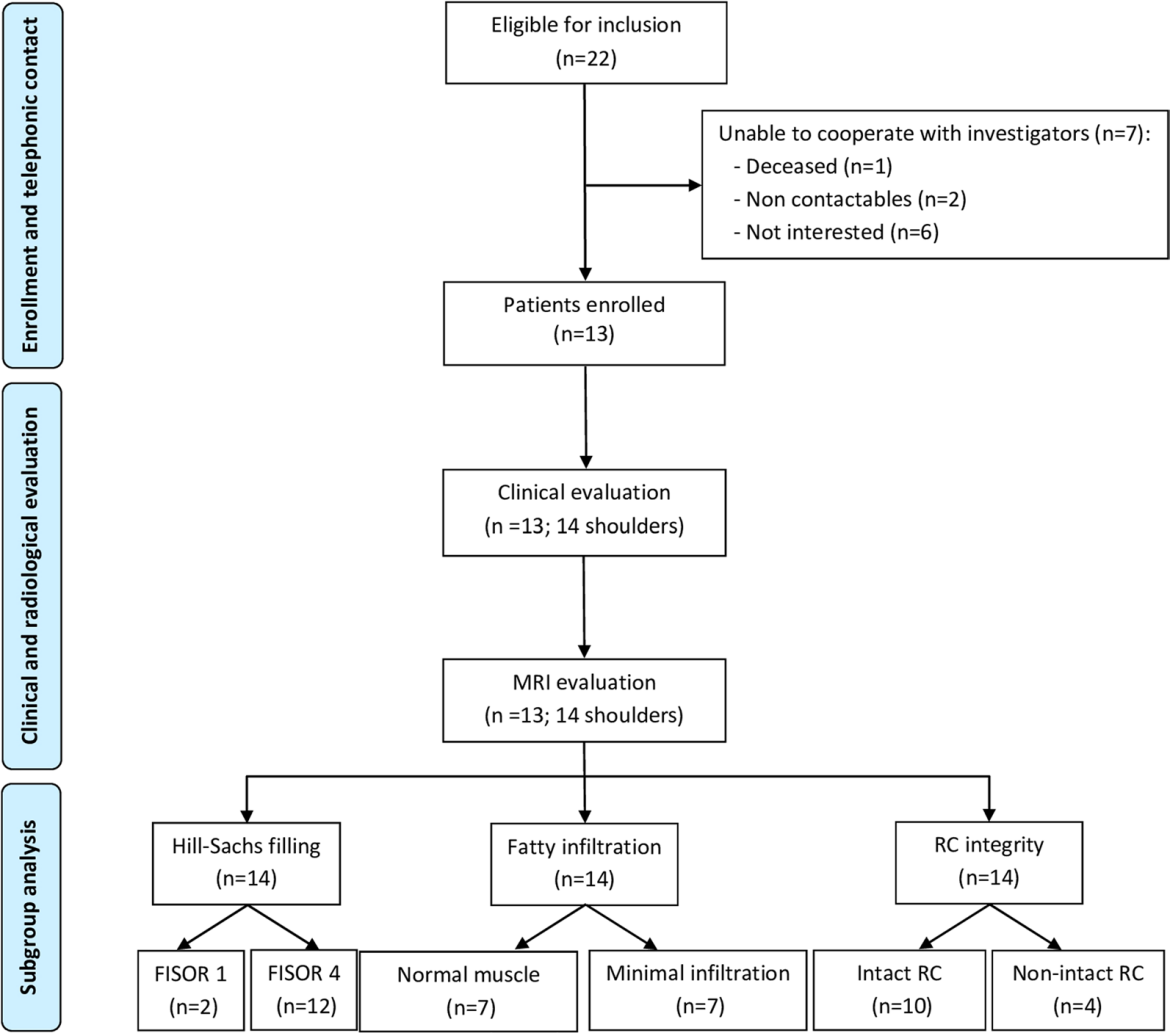
Flowchart of the study. *FISOR* Filling Index Score of Remplissage, *MRI* magnetic resonance imaging, *RC* rotator cuff

After applying the inclusion and exclusion criteria, 13 patients (14 shoulders) were eligible for clinical and radiological evaluation. The flow diagram shown in Fig. 2 illustrates the grouping and flow of patients in our clinical study. Demographic data for all the patients are reported in Table 2.

**Table 2:** Patient demographics

Study population	Overall
No. of patients (no. of shoulders)	13 (14)
Age at follow-up, years	29.00 (± 7.93)
Follow-up, months	55.93 (± 18.16)
F/M ratio	0.21/0.79
Dominant side (Y/N ratio)	0.36/0.64

Data are reported as mean (± SD), median [Q1–Q3], or frequency/ratio. *F/M* female/male, *No.* number, *Q1* first quartile, *Q3* third quartile, *SD* standard deviation, *Y/N* yes/no

All the shoulders examined underwent arthroscopic Bankart repair associated with Hill–Sachs remplissage performed using only one double-loaded suture anchor (Corkscrew 5.0 mm, Arthrex, Naples, FL, USA). Three shoulders also underwent a SLAP lesion repair, and one was subjected to an extra-articular tenodesis of the long head of the biceps.

The dislocation affected the non-dominant limb in 64.28% of the cases and occurred during a traumatic event in all the patients. Eleven patients (84.6%) practiced contact sports at a high injury risk (rowing, CrossFit, and volleyball). The rate of return to sport was 100%; only two patients decreased their level of performance in the same sport, and one changed sport.

Two patients (14.28%) reported a recurrence of instability after a trauma occurred during sporting activity (43 and 86 months after the surgical procedure, respectively). The surgeon proposed a Latarjet procedure in one patient and conservative treatment in the other.

One patient complained of persisting deep posterior/superior shoulder pain exacerbated by extreme abduction and external rotation movements, with occasional apprehension.

At the 3-year follow-up, all patients showed good clinical and functional outcome scores: the Walch–Duplay Score was 95.00 [87.25–100.00] points, the CMS was 87.36 (± 5.37) points, and the ASES was 97.50 [87.25–100.00] (Table 3).

**Table 3:** Summary of the main clinical and functional results at 3 years of follow-up

Study population	Overall
No. of patients (no. of shoulders)	13 (14)
CMS: 0–100 points	87.36 (± 5.37)
ASES: 0–100 points	97.50 [87.25–100.00]
Walch–Duplay: 0–100 points	95.00 [80.00–100.00]
ROM ABD, degrees	166.3 (± 12.16)
ROM FWF, degrees	175.00 [162.50–180.00]
Strength ABD, lbs	18.08 (± 4.44)

Data are reported as mean (± SD), median [Q1–Q3], or frequency/ratio. *ABD* abduction, *ASES* American Shoulder and Elbow Surgeons Score, *CMS* Constant–Murley Score, *FWF* forward flexion, *lbs* pounds, *Q1* first quartile, *Q3* third quartile, *ROM* range of motion; *SD* standard deviation

ROM evaluation revealed a mean abduction of 166.3° (± 12.16°) and a median forward flexion of 175.00° [162.50–180.00°].

Median values in external rotation of the affected limb, measured with the arm at the side (ER1) and with the arm abducted at 90° (ER2), were found to be 75.00° [70.00–75.00°] and 80.00° [80.00–80.00°], respectively.

Analysis of the data showed a significant median reduction in active external rotation ROM in the affected shoulders compared to the contralateral healthy ones in both ER1 (75.00° vs. 85.00°; *p* = 0.0005) and ER2 (80.00° vs. 90.00°; *p* = 0.0010) (Table 4).

**Table 4:** Comparison of ROM and the isometric strength of the infraspinatus between the affected shoulder and the contralateral healthy side

Groups	Affected shoulder	Healthy contralateral shoulder	*p*-value
No. patients (no. of shoulders)	12 (12)	12 (12)	
ROM ER1, degrees	75.00 [70.00–75.00]	85.00 [80.75–85.00]	0.0005
ROM ER2, degrees	80.00 [ 80.00–80.00]	90.00 [85.75–90.00]	0.0010
Strength ER1, lbs	12.08 (± 3.13)	12.84 (± 3.11)	0.2375 (n.s.)
Strength ER2, lbs	9.43 [8.44–14.91]	12.70 (± 4.05)	0.0342

Data are expressed as mean (± SD) or median [Q1–Q3]. *ER1* external rotation with the arm at the side, *ER2* external rotation with the arm at 90° of abduction, *lbs* pounds; *n.s.* not significant, *Q1* first quartile, *Q3* third quartile, *ROM* range of motion, *SD* standard deviation

Reductions in the ER1 strength and ER2 strength of the affected shoulders were found. However, the reduction was only statistically significant for ER2 (*p* = 0.0342) (Table 4).

Despite the loss of ROM and strength in external rotation of the affected shoulders, 77% of the patients reported satisfaction with their sporting and recreational ability after the procedure.

**Fig. 3:**
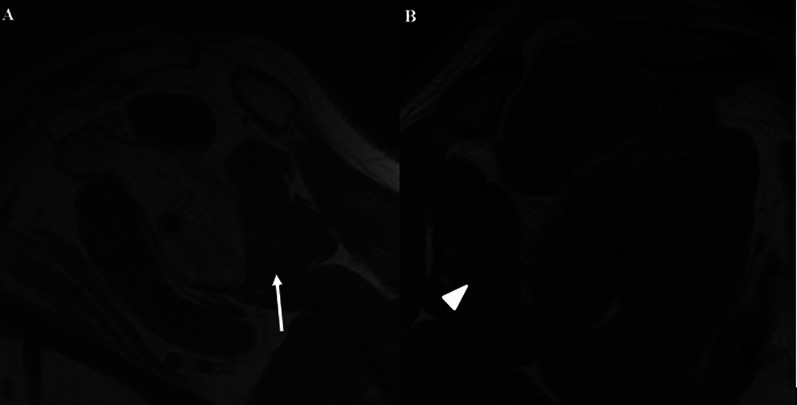
MRI sagittal cuts of the shoulder for two patients who underwent the remplissage procedure: **A** the* white arrow* reveals mild fatty infiltration; **B** the* arrowhead* shows the absence of fatty streaks inside the belly of the infraspinatus muscle

MRI evaluations of 14 shoulders revealed an absence of infraspinatus fatty infiltration in 50% of cases and a mild amount (Fuchs grade 1) in the remaining 50% (Fig. 3).

**Fig. 4:**
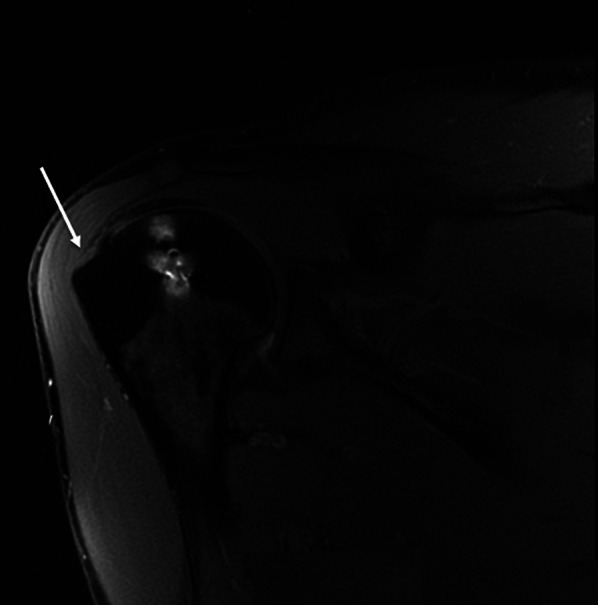
MRI coronal cut of the shoulder of a patient who underwent the remplissage procedure: the* white arrow* shows a partial tear, articular and bursal, of the supraspinatus tendon

Full-thickness rotator cuff tears and other degenerative manifestations involving the infraspinatus tendons were not observed. An area of delamination on both the articular and bursal sides of the supraspinatus tendon (type A2B2 according to the Snyder classification) was found in the two shoulders that underwent recurrence of dislocation (Fig. 4); mild synovial irritation of the supraspinatus (type A1 according to the Snyder classification) was detected in two others. These findings were not seen on preoperative MRI scans.

Signs of bone edema or resorption cysts at the humeral head were not noticed.

The measurements of the Hill–Sachs lesions highlighted a mean volume of 1060 (± 477.3) mm^3^.

**Fig. 5:**
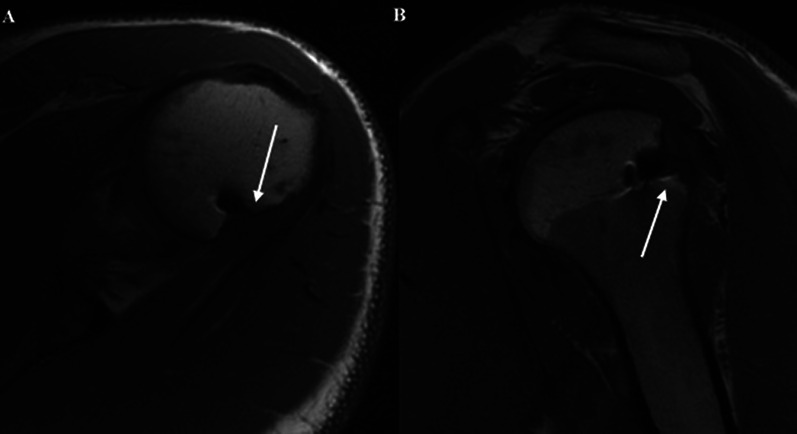
MRI axial **A** and sagittal **B** cuts of the shoulder of a patient who underwent the remplissage procedure. The* white arrows* show complete filling of the Hill–Sachs lesion in both the axial **A** and the sagittal **B** cuts

**Fig. 6:**
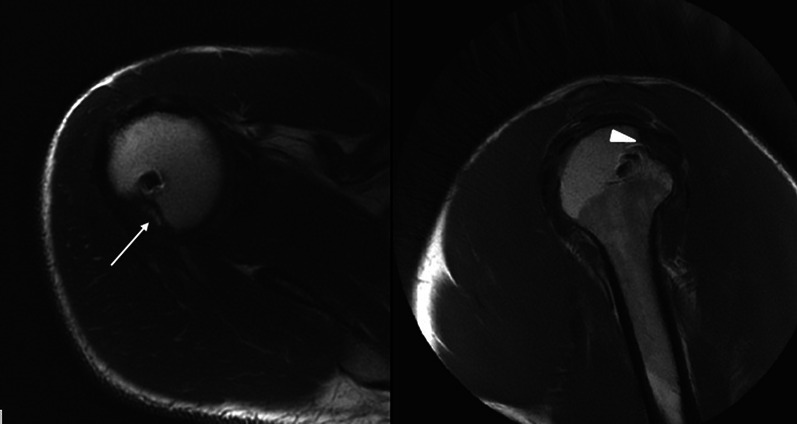
MRI axial **A** and sagittal **B** cuts of the shoulder of a patient who underwent the remplissage procedure: **A** the* white arrow* shows a fat tissue layer localized between the tendon and the bottom of the Hill–Sachs lesion; **B** the* arrowhead* reveals the partial filling of the bone defect

In 11 shoulders (78.57%), the concavity of the lesion was completely filled by the capsulotenodesis (FISOR grade 4) (Fig. 5); in one case (7.14%), it was 75–100% filled (FISOR grade 3). A layer consisting of mature fibrous tissue appeared at the tendon–bone interface in these shoulders. In the two recurrences, the infraspinatus tendon partially covered the defect (FISOR grade 1) and a layer of adipose tissue associated with unripe and poorly dehydrated fibrous tissue was found between the tendon itself and the humeral head (Fig. 6).

## Discussion

The main finding of this study is that MRI evaluation revealed the complete absence of fatty infiltration in half of the shoulders and only minimal infiltration in the remaining half (Fuchs grade 1), demonstrating the positive and effective biological response of the tissues, and thus confirming the hypothesis that remplissage is a safe procedure which, over the years, does not lead to pathological changes in the involved structures. MRI was chosen to detect the appearance of fatty infiltration in the context of the infraspinatus muscle due to the high-quality scans produced, which allowed for more accurate detection of fatty infiltration and an evaluation of the quality of the scar tissue placed at the tendon–bone interface.

High-grade filling (FISOR grade 3 or 4) of the Hill–Sachs lesion was successfully observed in 12 shoulders (85.71%), with hypointense healthy dehydrated fibrous scar tissue appearing between the infraspinatus tendon and the humeral head, demonstrating the healing of the capsulotendodesis. In the two cases of recurrence, the filling of the Hill–Sachs was < 25% (FISOR grade 1).

These results are in agreement with those reported by Park et al. [20], who noticed a complete absence of consistent fatty infiltration in MRI scans of 11 shoulders subjected to remplissage (with a mean Hill–Sachs volume of 334.3 mm^3^, significantly shorter than that reported in our study) during a mean follow-up of 18 months (minimum follow-up: 8.8 months). A time period of 18 months is probably too short to detect early signs of degeneration and assess the complete healing of the capsulotenodesis; in fact, Park et al. found the presence of granulation tissue in 72.72% of cases, and this finding seems to demonstrate that the healing process of those shoulders was still ongoing.

This finding is similar to that reported by Boileau et al. [23], who performed a radiological evaluation of 42 patients (CT arthrography in 38 patients and MRI in 4 patients; mean follow-up: 24 months) who underwent the remplissage procedure. Those authors highlighted capsulotenodesis healing in all the patients and noted that the Hill–Sachs lesions were more than 75% filled in 73.8% of the patients. This result, which was based on a larger sample size, confirms that filling the Hill–Sachs lesion is an essential condition to obtain healing of the tissue; however, the use of CT as the primary imaging device could represent an inherent bias of this study, as it is not the best choice to evaluate the quality of scar tissue.

Radiological evaluation of the two recurrences (14.29%) in the present study revealed significantly larger Hill-Sachs volumes (1963.9 mm^3^ and 2049.32 mm^3^, respectively) and adipose tissue located at the tendon-bone interface of the capsulotenodesis, suggesting that complete filling could be essential for the healing process.

The use of a single metallic anchor in Hill–Sachs of this size might cause recurrences. Still, it is impossible to know precisely if the larger size of the Hill–Sachs lesion, the tendon detachment, and the appearance of fatty tissue were direct consequences of the new trauma or were previously present.

No complete thickness rotator cuff tears were identified in any of the shoulders upon MRI examination, and the infraspinatus tendon was completely intact, showing no signs of fraying or irritation. Supraspinatus tendon partial tears were found in four shoulders, with the larger ones (A2B2 according to Snyder’s classification) affecting the two recurrences. A possible explanation for these findings could be the uncoupling between static and dynamic stabilizers in these two shoulders following the stabilization failure, with eccentric tensile forces causing significant stress at the supraspinatus tendon and, over time, a consequent tear [24].

The postoperative clinical and functional evaluation showed good-to-excellent outcomes; in particular, the Walch–Duplay Score (95.00 [80.00–100.00]) was excellent in nine shoulders (64.28%), good in four shoulders (28.67%), and medium in one shoulder (7.14%). Similar results were also reported by Merolla et al. [25] based on 61 remplissage procedures; good functional outcomes were maintained, with a mean CMS of 90 points and a mean Walch–Duplay Score of 90.4 after a mean follow-up of 39.5 months.

A relevant finding of this study is the statistically significant loss (median: 10°) of ROM in external rotation, both in ER1 (*p* = 0.0005) and in ER2 (*p* = 0.0010), compared to healthy contralateral shoulders. These findings follow those reported by Boileau et al. [23], who referred to mean deficits of 8° (± 7°) in ER1 (*p* < 0.001) and 9° (± 7°) in ER2 (*p* < 0.001), and Merolla et al. [25], who found a significantly lower ER1 (68.3° vs. 89.1°, *p* < 0.001) and ER2 (74.1° vs. 89.4°, *p* < 0.001) compared to healthy shoulders. On the contrary, Garcia et al. [26] did not detect significant differences in postoperative ER2 (83.95° vs. 89.21°,* p* = 0.13) compared to the contralateral shoulders, and Franceschi et al. [27] found only a minimal reduction in postoperative ER1 (56.0° vs. 60.6°, *p* = 0.4) compared to the preoperative assessment. The latter authors also compared the postoperative external rotation reached after Bankart repair and remplissage to that achieved following Bankart repair alone and did not find any statistically significant differences (*p* = 0.02).

Isometric infraspinatus strength testing revealed superior strength of the contralateral healthy shoulder, and we found that only ER2 showed a statistically significant decrease (*p* = 0.0342). Merolla et al. [25] were first to assess the infraspinatus strength: the measurement was performed with the arm at the side and with the examiner standing in front of the patient and resisting the external rotation force. They did not find a significant reduction in ER1, but they did not measure ER2. This type of measurement with the examiner resisting the force of the patient could be less precise and more variable than that achieved with the dynamometer stably fixed to a post, so the results obtained could have been influenced by the strength exerted by the examiner himself.

However, none of the patients included in the present study had a loss of ROM or strength that affected their daily and sports activities, as reported by Buza et al. [24] in a systematic review of six clinical studies (167 patients) with a mean follow-up of 26.8 months. No ROM reduction, especially in ER1, was perceived by our patients. In ER2, a feeling of tension when the patient or the examiner brought the arm to the maximum angle of abduction external rotation was sometimes referred to. The patients did not perceive a significant limitation in strength at any degree of abduction (despite the appearance of minimal signs of adipose atrophy in the context of its muscular belly in 50% of the shoulders).

The rate of return to sport was 100%, with 92.3% returning to their previous sport and 76.92% returning to their pre-injury level, which are similar results to those of Boileau et al. [23] and Garcia et al. [26].

The recurrence rate of 14.28% is higher than in some other studies (0% [28], 4.4% [29], 9.1% [26]) but similar to that reported by Park et al. [30], who noted a 15% recurrence rate at a mean follow-up of 30 months. In a recent retrospective study, Pandey et al. [31] compared the recurrence rates of off-track Hill–Sachs lesions treated with and without the use of remplissage, and found a significantly lower recurrence rate of 3.4% in the remplissage group compared with the Bankart repair alone group (30%). These findings support the hypothesis that the remplissage procedure, in association with Bankart repair, is effective and reliable for treating anterior shoulder instability. It is also safe, with no appearance of fatty degeneration of the infraspinatus muscle or other complications, and yields a low recurrence rate. It brings a loss of ROM and a reduction of strength in external rotation that are not a source of discomfort for the patients.

This study's strength is that the same surgeon performed all the procedures and all the patients underwent the same postoperative rehabilitation protocol. The cohort presented in this study, with a mean follow-up of 55.9 months, is the second for which such long-term results are described [23, 26–31].

Patients were chosen with a maximum age of 45 years to exclude those who could already have degenerative changes of the rotator cuff at the preoperative level. The Hill–Sachs lesions in older patients are usually more extensive due to poorer bone quality. Additionally, these patients have lower shoulder function and lower functional request than younger patients. All of this could have led to a bias in the analysis of the results.

Limitations of this study include the small sample size due to the infrequent indication for this surgical procedure and the relatively high rate of patients who were unwilling or unable to return to the institution for clinical evaluation.

A measurement of the contralateral healthy shoulder was conducted to compare the results to the affected one. The fact that preoperative ipsilateral measurements were not available could have represented a slight bias in the results. However, the discrepancy between the affected side and the contralateral side could have arisen because patients did not perform the complete extra rotation of the arm preoperatively due to a feeling of apprehension that the shoulder was experiencing subluxation or dislocation, as recently reported by Pandey et al. [31].

Furthermore, the measurement of the infraspinatus strength in this study could have also been influenced by the fact that eight of the shoulders (66.66%) represented the non-dominant limb, which may have distorted the side-to-side comparison, as they would have naturally been less intense than the dominant one.

## Conclusions

Arthroscopic remplissage, despite the increased cost and longer surgical time, is a reliable and reproducible procedure for treating shoulder instability associated with a significant humeral bone defect; it is also safe in the medium-to-long term, as it does not cause degenerative effects in involved structures and it reduces the recurrence rate when compared to isolated Bankart repair.

## Data Availability

Data and materials generated during the current study are available from the corresponding author on reasonable request.
